# ‘It feels like a blessing’ – The experience of Hebrew-English bilingualism among autistic children: An interpretative phenomenological analysis

**DOI:** 10.1177/13623613251367244

**Published:** 2025-09-15

**Authors:** David Ariel Sher, Nicole Wendy Sher, Jenny L Gibson, Hannah Ella Sher

**Affiliations:** 1 University of Oxford, UK; 2 University of Cambridge, UK; 3 The University of Manchester, UK

**Keywords:** autism, autistic, bilingualism, Hebrew, Jewish, qualitative

## Abstract

Over half the world’s population are bilingual. Despite this, practitioners often advise parents of autistic children to teach their child one dominant national language and limit their child’s exposure to community languages. Limited research with autistic children’s carers has shown that this monolingual approach impoverishes children’s experiences in communal, familial, religious, and social contexts. There is very little research on this topic from the first-person perspectives of autistic children themselves. There is no research exploring the perspectives of Hebrew-English bilingual autistic children. This study aimed to explore the perspectives and experiences of autistic children on Hebrew-English bilingualism. In accord with the interpretative phenomenological analysis (IPA) methodological framework, semi-structured interviews were conducted to explore the experiences of 13 Hebrew-English bilingual autistic children. The IPA clustering process resulted in two overarching group experiential themes (GETs). The first GET was ‘Bilingualism aids religious, educational, and social integration and connection’. The second GET was ‘Preference of bilingualism and dislike of monolingual approach’. Recommendations include training for practitioners regarding potential positive outcomes of bilingualism, alongside the way their advice to adopt a monolingual approach impacts upon autistic children. We also argue the importance of autistic children’s views being considered whenever bilingual decision-making is made.

**Lay abstract:**

Over half the world’s population speak two or more languages. Despite this, practitioners often advise parents of autistic children to teach their child one main national language and not expose their child to additional community languages. Limited research with autistic children’s carers has shown that this approach negatively impacts autistic children’s communal, family, religious, and social experiences. There is very little research on this topic from the first-hand perspectives of autistic children themselves. There is no research exploring the perspectives of autistic children who speak both Hebrew and English. This study aimed to explore the views of autistic children who have ability in the Hebrew and English languages. Using the interpretative phenomenological analysis (IPA) research approach, we conducted interviews to explore the experiences of 13 autistic children who have ability in both the Hebrew and English languages. The research resulted in two overarching themes. The first theme was ‘Bilingualism aids religious, educational, and social integration and connection’. The second theme was ‘Preference of bilingualism, and dislike of monolingual approach’. Our recommendations include training practitioners to be more aware of the way the advice they give for autistic children to only learn one language can negatively impact autistic children. We also argue that whenever decision-making about an autistic child learning two or more languages is made, autistic children’s views should be considered.

Autism is a lifelong neurodevelopmental condition whose clinical features were first outlined by Grunya Efimovna Sukhareva, a Russian psychiatrist, in 1925 (Sher & Gibson, 2023). According to conventional clinical diagnostic criteria, autism is characterised by repetitive or restricted behaviour patterns and challenges in social interaction and communication (American Psychiatric Association, 2013). More recent clinical perspectives on autism emphasise autistic strengths and the role of the environment in any presenting challenges (Pritchard-Rowe & Gibson, 2024). In the present article, we consider the linguistic and cultural aspects of the environment and their impact on Jewish, autistic children.

Forced monolingualism refers to being prevented from gaining ability in an additional language despite the language being important to one’s culture or family (Clyne, 1991, 2005; Sher, 2022). A propensity among practitioners to advise parents to limit an autistic child to one language has been observed (Beauchamp & MacLeod, 2017; Drysdale et al., 2015; Gréaux et al., 2020; Hampton et al., 2017; Jegatheesan, 2011; Kay-Raining Bird et al., 2012; Kremer-Sadlik, 2005; Ohashi et al., 2012; Uljarević et al., 2016). However, there is very little empirical research to back such a stance (Uljarević et al., 2016). Indeed, forced monolingualism negatively impacts autistic children, partly by impeding communal integration and social opportunities, and by infringing on children’s basic communicatory rights (Gréaux et al., 2020; Uljarević et al., 2016). Autistic children from backgrounds where learning Hebrew is important may be impacted by this approach. Hebrew occupies a central role in the lives of millions of Jews worldwide (Glinert, 2017). It is deemed crucial for the continued existence of the Jewish identity (Mintz, 1993; Schiff, 1998). However, there is sparse research on this topic, and no study has explored this topic from autistic children’s perspectives. We therefore set out to understand autistic children’s perspectives and experiences of Hebrew-English bilingualism.

In this study, we accept the definition of a multilingual being a person who is able to speak more than one language, and a bilingual being an individual with ability to speak two languages (Goral & Conner, 2013).

## The impact of forced monolingualism

Over half the world’s population speak two or more languages (Grosjean, 2021). Thus, a ubiquitous proclivity to limit autistic children’s exposure to one language could have an impact on a widespread scale. Kay-Raining Bird et al. (2012) found only 12.5% of 49 parents of autistic children were advised to encourage their child’s proficiency in an additional language; 62.5% were recommended they should not (Kay-Raining Bird et al., 2012). This is mirrored in many other studies (Beauchamp & MacLeod, 2017; Drysdale et al., 2015; Hampton et al., 2017; Jegatheesan, 2011; Kay-Raining Bird et al., 2012; Kremer-Sadlik, 2005; Ohashi et al., 2012; Uljarević et al., 2016). Reasons to explain this monolingual tendency are often based on practitioner intuition rather than research evidence. Such assumptions can include that two languages for autistic children is ‘too much’, or too ‘confusing’ (Gréaux et al., 2020), or that since some autistic children may find learning one language difficult, assumedly learning two will be even harder (Ijalba, 2016; Lim et al., 2018). There are also concerns from practitioners about language and literacy progression if autistic children learn two languages (Howard et al., 2024). Yet reviews have found that bilingualism causes no such language delays and does not impede language acquisition (Garrido et al., 2024; Uljarević et al., 2016). In fact, research suggests bilingual autistic children may enjoy social and cognitive benefits (Beauchamp & MacLeod, 2017; Dai et al., 2018; Genesee et al., 1996; Gross & Rutland, 2022; Lund et al., 2017), including enhanced control over motor impulsivity (Montgomery et al., 2022), performing better than monolingual counterparts on set-shifting tasks (Gonzalez-Barrero & Nadig, 2019), on total expressive vocabulary (Petersen et al., 2012; Seung et al., 2006), and being more likely to gesture and vocalise than monolingual peers (Valicenti-McDermott et al., 2013). When children become bilingual and able to speak a parent’s primary language, this enhances parent-child relationships (Hampton et al., 2017). A study with 75 autistic children found no language delays associated with bilingualism (Hambly & Fombonne, 2014). Emerging research suggests that practitioners are not made aware of research findings such as these, which in turn impedes their ability to provide parents with evidence-based advice on autism and bilingualism (Davis et al., 2024).

When children of any background learn a language important to their cultural or ethnic heritage, this often strengthens communal connection (He, 2010; Müller et al., 2020), and enhances cultural cohesion and social opportunities (Creese et al., 2006). When autistic children’s parents are told to prevent their child’s exposure to a second language, this limits children’s vocabularies, and impedes maintenance of heritage languages (Fahim & Nedwick, 2014). It can also prevent valuable opportunities for social and communal integration in familial, religious, or other community contexts (Jegatheesan, 2011; Kremer-Sadlik, 2005; Sher, 2022; Sher, Gibson, & Browne, 2022). A monolingual approach also often runs counter to parents’ wishes (Howard, Gibson, et al., 2021; Howard, Katsos, et al., 2021; Kay-Raining Bird et al., 2012; Yu, 2013) and causes familial tension, as children have less understanding of cultural norms within their family (Howard et al., 2019a; Jegatheesan et al., 2010; Yu, 2013). For reasons such as these, a recent policy paper declared that ‘*autistic children with multilingual backgrounds*’ face an ‘unacceptably high’ risk of ‘*all aspects of their communication rights being violated*’ (Gréaux et al., 2020).

## Importance of Hebrew

Hebrew is among the most ancient of languages, preceding both Greek and Latin in its antiquity (Aviram, 1974). The Hebrew language originates largely from the Hebrew Bible, which, following translation into every language, is argued to be ‘*the most globally influential and widely read book ever written*’ (Huguelet & Koenig, 2009, p. 31). For Jews, Hebrew is the language of religious law, prayers and scripture, and in recent times, it is the language of the modern state of Israel (Glinert, 2017). Judaism’s continuity has been argued to be entwined with the maintenance of Hebrew (Gross & Rutland, 2022; Mintz, 1993; Schiff, 1998). Perhaps in recognition of this, Hebrew is an important subject in Jewish schools worldwide (Gross & Rutland, 2014; Kraemer & Zisenwine, 1989; Miller, 2011; Raijman, 2013; Schick, 2009). There are specialist Jewish schools in several countries that cater for autistic children. Being able to speak Hebrew is important for Jewish autistic children, to enable participating in religious life and mark lifecycle events, including *bar*/*bat mitzvah* (Hyman, 2009; Muskat & Putterman, 2016).

## The need for the present study

However, there is very limited research on bilingualism from the first-person perspective of autistic children in general (Howard et al., 2019a), and to our knowledge, no research specifically on autistic children’s perspectives on Hebrew-English bilingualism. One study has been conducted on the school experiences of 11 autistic children (aged 7–14) living in England and Wales (Howard et al., 2019a). This found that while autistic children in monolingual school settings appeared uncomfortable with being identified as bilingual, those in more multilingual settings tended to embrace their bilingualism and noted the positive outcomes that bilingualism afforded them (Howard et al., 2019a). Moreover, it was found that participants did not tend to describe themselves as being ‘different’ to their peers, even though, in contrast to their peers, they were both autistic and bilingual (Howard et al., 2019a). One study has explored perspectives of parents and educational practitioners on Hebrew–English bilingualism for autistic children (Sher, Gibson, & Browne, 2022). In accord with previous research (Hampton et al., 2017), the study found that non-Jewish paediatricians and speech therapists advised against bilingualism for autistic children. Conversely, unlike research findings that educators doubted the feasibility of bilingualism for autistic children (Howard et al., 2024), the study found that Jewish practitioners and families championed autistic children gaining Hebrew-English proficiency. This may align with recent findings indicating the United Kingdom (UK) Jewish community has made progress in overcoming stigma towards autism (Sher, Gibson, & Sher, 2022). In contrast to other research (Kremer-Sadlik, 2005; Yu, 2013), due to support for Hebrew-English bilingualism among Jewish schools and practitioners, parents encouraged their children to gain Hebrew-English proficiency (Sher, Gibson, & Browne, 2022).

In general, the voice of autistic children has been insufficiently presented within research (DePape & Lindsay, 2016; Tesfaye et al., 2019). The present research answers general calls for further research on autism and bilingualism (Davis et al., 2022), for research focussed on the perspectives of autistic children (DePape & Lindsay, 2016), for research within understudied cultures and ethnic minorities (Jegatheesan et al., 2010), and for more research with autistic children on bilingualism specifically (Howard et al., 2019a). The aim of the present study was to address the research question ‘*What are the views of autistic children on Hebrew-English bilingualism?*’

## Method

### Design

This is a qualitative study that used the interpretative phenomenological analysis (IPA) methodological framework. IPA is suited for exploratory research on understudied topics (Smith et al., 2022). IPA is also apposite for the research focus of this study, as it views participants as experts on their experiences (Smith et al., 2022) and so can level power imbalances between autistic participants and researchers (Howard et al., 2019b; MacLeod et al., 2018). Moreover, IPA’s unique features, including the doubly reflexive ‘double hermeneutic’ (Smith et al., 2022), can help address the ‘double empathy’ problem (see Milton, 2012) which so often blights autism research (Howard et al., 2019b). This was achieved, in part, through the researchers in this study placing autistic participants at the heart of sense-making of their experiences, and through the greater reflexivity the researchers had to engage in as part of the double hermeneutic (whereby one cannot hurriedly assume that one has truly grasped what the participant means; Howard et al., 2019b).

### Researcher reflexivity

The first author is an ordained rabbi; holds degrees in Jewish education, education and psychology; has completed IPA and qualitative training, and is part of a phenomenological research group. The second author is an educational psychologist, holds degrees in psychology and educational psychology, has completed IPA and qualitative training, and works within several UK Jewish schools. The third author is a professor of neurodiversity and developmental psychology, holds qualifications in psychology and speech and language therapy, has received qualitative research training, and has personal and professional experience of neurodevelopmental difficulties. The fourth author has completed qualitative research training, holds degrees in psychology and clinical and health psychology, and works within UK Jewish schools. A reflexive approach was employed throughout this study. A key way of engaging reflexively in qualitative work is through reflexive writing (Olmos-Vega et al., 2023). Thus, the first author (who conducted primary analysis) completed reflexive memos, which are deemed a key way to enhance reflexivity (Birks et al., 2008; McGrath, 2021). A ‘bridling’ approach was adopted (Dahlberg, 2006). Through this, in accord with the thought of Merleau-Ponty (Dahlberg, 2006; Merleau-Ponty, 2002), rather than trying to excise past experiences and knowledge (which is both improbable and undesirable; Dahlberg & Dahlberg, 2020), the researcher reflected on these to enable greater self-awareness and an ability to be open to new understandings (Dahlberg & Dahlberg, 2019).

Bridling involves exercising control over one’s assumptions and learning made from previous experiences so that they do not cause one to miscomprehend new phenomena, nor lead one into understanding these too narrowly (Dahlberg, 2006). Bridling involves reflection and consideration of one’s presuppositions and requires the researcher to be aware of ways in which these might impact on one’s understanding of new concepts (Dahlberg & Dahlberg, 2020). It encourages being receptive to new concepts and thoughts but directs this openness so that one does not entirely discard one’s past experiences, which can be useful frameworks to understand new ideas (Dahlberg and Dahlberg, 2020).

### Participants

This study is part of a project based at the University of Cambridge which explores experiences of Jewish autistic children. Purposive, homogeneous sampling was used for recruitment. In accord with patient and public involvement (PPI) advice from an expert by experience who is a member of the autism community (please see ‘Community involvement’ section further within this article), the study was advertised using Facebook accounts popular with the autism community in the UK and Israel. This accords with findings that parents of autistic children rely on Facebook for support and information (Mohd Roffeei et al., 2015), and that Facebook is a highly effective platform for recruitment in autism research (Ahmed et al., 2020). Parents contacted the first author by email to express interest in taking part. The first author explained the study to interested parents and provided them with written information about the study and a consent form. Twenty-four hours had to elapse to consider participation before interview arrangements were made. Parents and participants could decline participation at any point. Based on recommended sizes for IPA research, 13 participants were interviewed. The sample size accords with previous IPA autism research (Howard et al., 2019b) and aligns with IPA’s idiographic commitment, which requires smaller sample sizes (Nizza et al., 2021; Smith et al., 2022). Participants’ ages ranged between 8.5 and 17 years (*M* = 11.6, standard deviation = 2.1). All participants had an autism diagnosis, and nine had comorbidities (attention deficit hyperactivity disorder, *n* = 7; anxiety, *n* = 2; 22q11.2 deletion syndrome, *n* = 1). Clinical and demographic information appears in Table 1. The clinical and demographic information presented in Table 1 was provided by parents in telephone calls that were held prior to interviews taking place.

**Table 1.** Participant information.

Participant pseudonym^a^ and country	Age	Gender	Diagnosis and comorbidities	Jewish denomination child belongs to	School strand of Judaism	Languages	Proficiency in languages
**Yael** North-West England	12 years	Female	Autism spectrum condition, 22q11.2 deletion syndrome	Centrist or Modern Orthodox	Centrist or Modern Orthodox	English, Hebrew	English: reads, speaks, understands, and writes English very well. Hebrew: reads and writes Hebrew well. Cannot speak or understand Hebrew.
**Aviva** North-West England	17 years	Female	Autism spectrum condition	Strictly Orthodox	Strictly Orthodox	English, Hebrew	English: reads, speaks, and understands English very well. Writes English well. Hebrew: writes and reads Hebrew well. Struggles to speak and understand Hebrew.
**Shalom** Jerusalem district, Israel (3 years), formerly South-East England (7 years)	9 years	Male	Autism spectrum condition, ADHD	Centrist or Modern Orthodox	Centrist or Modern Orthodox	English, Hebrew	English: reads, speaks, understands, and writes English very well. Hebrew: speaks and understands Hebrew very well. Struggles to write and read Hebrew.
**Tiffany** North-West England	10 years	Female	Autism spectrum condition	Strictly Orthodox	Strictly Orthodox, specialist SEND	English, Hebrew	English: speaks and understands English very well. Reads and writes English well. Hebrew: understands Hebrew well. Struggles to write, speak, and read Hebrew.
**Conrad** South-East England	11.5 years	Male	Autism spectrum condition, ADHD	Traditional/ Modern Orthodox	Centrist or Modern Orthodox	English, German, Hebrew	English: reads, speaks, understands, and writes English very well. German: reads, speaks, understands and writes German well. Hebrew: writes and reads Hebrew well. Struggles to speak and understand Hebrew.
**Yoel** North-West England	8.5 years	Male	Autism spectrum condition, ADHD	Hasidic/Strictly Orthodox	Strictly Orthodox, specialist SEND	English, Hebrew, Yiddish	English: speaks and understands English very well. Cannot read or write English. Yiddish: understands Yiddish very well. Speaks Yiddish well. Cannot read or write Yiddish. Hebrew: struggles to read Hebrew. Cannot write, speak, or understand Hebrew.
**Saul** Central Israel (5 years), formerly South-East England (6 years)	11.5 years	Male	Autism spectrum condition	Secular	Israeli non-denominational school	English, Hebrew	English: reads, speaks, and understands English very well. Writes English well. Hebrew: reads, speaks, understands, and writes Hebrew very well.
**Kfir** Central Israel	11 years	Male	Autism spectrum condition, ADHD	Secular	Israeli non-denominational school	English, Hebrew	English: reads, speaks, and understands English very well. Struggles to write English. Hebrew: speaks, reads, and understands Hebrew very well. Struggles to write Hebrew.
**Jonah** South-East England	12.5 years	Male	Autism spectrum condition	Traditional	Non-Jewish, specialist SEND school	English, Hebrew	English: speaks and understands English very well. Struggles to read and write English. Hebrew: reads, speaks, and understands Hebrew well. Struggles to write Hebrew.
**Yair** Jerusalem district, Israel (8 years) North-Eastern USA (4.5 years)	12.5 years	Male	Autism spectrum condition, ADHD, anxiety	Traditional	Israeli non-denominational school	English, Hebrew	English: reads, speaks, and understands English very well. Struggles to write English. Hebrew: reads, speaks, and understands Hebrew very well. Struggles to write Hebrew
**Devorah** Jerusalem district, Israel	11.5 years	Female	Autism spectrum condition, anxiety	Centrist or Modern Orthodox	Israeli religious specialist SEND school	English, Hebrew	English: speaks and understands English very well. Reads and writes English well. Hebrew: speaks and understands Hebrew well. Struggles to read and write Hebrew.
**Joshua** South-East England	10 years	Male	Autism spectrum condition, ADHD	Traditional	Centrist Orthodox school	English, Hebrew	English: reads, speaks, and understands English very well. Writes English well. Hebrew: reads, writes, speaks, and understands Hebrew well.
**Raphael** Jerusalem district, Israel	14 years	Male	Autism spectrum condition, ADHD	Centrist or Modern Orthodox	Centrist or Modern Orthodox school	English, Hebrew	English: reads, writes, speaks, and understands English very well. Hebrew: speaks and understands Hebrew well. Struggles to read and write Hebrew.

ADHD = attention-deficit hyperactivity disorder.

a Pseudonyms allocated by participants or participants’ guardians. Pseudonyms allocated by first author if participants or participants’ guardians did not indicate a preferred pseudonym.

### Inclusion criteria

Participants had to:

1. Have an autism spectrum condition (ASC) diagnosis;
2. attend school (primary or secondary school);
3. have proficiency (to any extent) in both Hebrew and English.

### Ethical approval

Ethical approval was applied for and ethical clearance granted by the institutional ethics committee. Participants were recruited through their parents, who discovered the study through adverts placed on social media platforms used by the autism community. The parents of all child participants, and one participant aged 17, provided written informed consent for study participation. Verbal assent to participate was gained from all child participants. Participants were offered the opportunity to choose a pseudonym for this study. Where no preference for a pseudonym was expressed by participants, a pseudonym was allocated by the first author. We were transparent about the aims of this research. Children were aware that the study concerned autistic children’s experience of Hebrew-English bilingualism. All children were also aware of their autism diagnosis prior to the interview taking place. This was unsurprising given literature indicating that a majority of parents choose to disclose their child’s autism diagnosis to them during childhood, and believe it should be discussed as soon as feasible (Crane et al., 2019, 2021). It is ethically important to ascertain that children are aware of their autism diagnosis prior to interviews, to avoid unwittingly disclosing an autism diagnosis to a child who was not formerly aware. This is especially so given the sensitivity and care which needs to accompany disclosing an autism diagnosis to a child (Crane et al., 2021; Smith et al., 2018, 2019).

### Procedure

The first, second, and fourth authors conducted semi-structured interviews between 2 June 2021 and 29 May 2023. Eleven interviews took place via video-call due to Covid-19 or participant preference, and two took place face-to-face. Interviews were audio-recorded and lasted between 17 and 52 minutes (*M* = 33.3, *SD* = 12.3). All interviews were conducted in English. Questions included ‘*Can you tell me which languages you can speak or read or write in?*’ and ‘*Do you like using Hebrew words?*’ In accord with the IPA autism research recommendation that perspectives of family members can provide valuable additional information to that provided by participants (Howard et al., 2019b), parents were allowed to add supporting perspectives during interviews, but in accord with Howard et al.’s (2019b) guidance, they were never allowed to ‘overshadow’ autistic children’s accounts. During interviews, it was ensured that children were able to give their view on any matters discussed by parents, by asking children about their perspectives on points that parents contributed. Children rather than parents were asked the interview questions, to help ensure that parents did not dominate interviews. Parents were able to add additional details or context if they wished. When annotating transcripts, parents’ accounts were useful for providing additional context, which accords with the guidance provided by Howard et al. (2019b). However, when parents’ contextual details differed from those provided by children, the children’s accounts were given primacy for annotation and interpretative coding. Potential prompts were included within the interview schedule, in recognition that these can be useful for research in this field (Harrington et al., 2014). In accord with methodological trends towards using multi-modal methods for eliciting perspectives of autistic children (Simpson et al., 2022), including photos or other visual cues (Tesfaye et al., 2019) or objects (Scott-Barrett et al., 2023), Jewish cultural objects with Hebrew names were shown in the interview, alongside photographs if required. These were used to ask participants if they knew the Hebrew name of these objects and to stimulate discussion and engagement within the interview. Interviews were adapted to facilitate participants engaging in a desk-based activity while they were talking (e.g. moving marbles on a board), where participants or parents indicated that this was helpful for regulation and concentration. Participants were also given the choice to engage with the interview in non-traditional ways if they preferred. For example, one participant drew and coloured objects related to the interview and described these to the interviewer, while another brought his shofar to the interview and sounded this to emphasise points made.

### Data preparation and analysis

Interviews were transcribed verbatim. Pauses, ‘guggles’, and ‘non-verbal utterances’ (Smith et al., 2022) were transcribed to add context. Identifying information was removed. Data analysis accorded with the IPA approach (Smith et al., 2009, 2022). The first and fourth authors read the transcripts several times. The first author conducted the coding. Annotations were made on each transcript, and experiential statements identified. In accord with the process outlined by Smith et al. (2022), experiential statements were written on paper, cut out, scattered, and laid across a flat surface for clustering. Patterns were then searched for, and experiential statements included in the pattern were moved so that they lay close together on the flat surface. Personal experiential themes (PETs) were then identified by clustering the experiential statements. Group experiential themes (GETs) were then formed by looking at all PETs, determining connections and commonalities across PETs, and then clustering these accordingly. All authors had the opportunity to review PETs and GETs, including wording of titles, and all authors agreed on the final themes presented within this manuscript.

### Quality of the research

The rigour of the study was enhanced by adapting interviews for autistic children (Scott-Barrett et al., 2023; Tesfaye et al., 2019), as outlined earlier. It was also enhanced by achieving markers of quality in IPA work, including forming a ‘compelling, unfolding narrative’, ‘developing a vigorous experiential account’, ‘close analytic reading of participants’ words’, and ‘attending to convergence and divergence’ (Nizza et al., 2021, p. 371; Yardley, 2000).

### Community involvement

The research was committed to working with autistic and Jewish communities to encourage meaningful rather than tokenistic input from these communities (Boylan et al., 2019). Four experts by experience from autistic and Jewish communities were consulted for input in this study. They advised on study design, recruitment, implementation, and how results should be interpreted. This strengthened the study. For example, one expert by experience suggested advertising the study via Facebook accounts which are popular with Israeli and UK-based autism communities. Another expert by experience from the autism community suggested that in Israeli contexts where Hebrew is the primary language, the interviewer should be encouraged to fully explore the participant’s relationship with English, as this is the language not spoken by all peers in many Israeli contexts. Other examples of changes made due to expert-by-experience suggestions, following their review of the text, included making several interpretative comments clearer within the results section by adding contextual information (e.g. providing further information about the context of Aviva’s General Certificate of Secondary Education (GCSE) experience). All researchers and participants had personal and professional experience of autism and/or were members of the Jewish community (indeed, one researcher is a rabbi). This promoted community-based engagement and focus. An accessible summary of this study will be provided to members of the autistic and Jewish communities following publication.

## Results

The first GET refers to the sense of integration and connection that participants explained bilingualism assisted them with. This integration and connection occurred in several domains, including at school, among peers, and in religious settings, all of which are outlined in the first GET. The second GET refers to participants’ explanation of bilingualism being unambiguously preferred, alongside their dislike of the notion of only knowing one language. It seems the preference for having Hebrew-English bilingual ability, and the dislike of monolingualism, was linked to reduced empowerment (which participants explained being monolingual would cause). In addition, participants explained that being monolingual could be burdensome for several reasons, which are elucidated in the second GET.

## GET: bilingualism aids religious, educational, and social integration and connection

The first GET reflects that often, participants explained that Hebrew-English bilingualism had assisted with integration and connection across several domains, including their religious, scholastic, social, and familial experiences.

### Communal integration

Participants relayed how bilingual ability aided social integration, often within communal settings. For instance, Yael reflected on participating in communal prayer

. . . we [children] used to go up to the bimah [synagogal platform] and sing . . . we did Yimloch [Hebrew prayer] [re: what she liked about it:] . . . I like that we all go up together like at Har Sinai [Mount Sinai]. (Yael, 12 years old, UK)

The threefold repetition of ‘we’ and phrasing ‘all go up together’ are seemingly used to reflect the strong communal and social meaning of this experience for Yael. By referring to Mount Sinai, Yael may be suggesting the shared experience felt religiously charged, akin to the revelatory Sinaitic experience.

### Social connection and integration

Raphael reflected on other social advantages of Hebrew-English bilingualism:

All my classes are in Hebrew. And my teachers just speak Hebrew. But I connect more with the Americans and those mainly my friends are mainly American. Yeah and culture that’s more similar . . . And also because my Hebrew isn’t fully fluent, it’s easy for me to like talk and socialise. (Raphael, 14 years old, Israel)

By framing the languages as providing access to two domains, Raphael emphasised the facilitative meaning that Hebrew-English bilingualism had for him. Knowing Hebrew meant scholastic engagement was possible, but ability in English meant connection and interaction with American classmates was enabled. It seems gaining Hebrew proficiency may have been challenging, as Raphael explained he was not entirely fluent in Hebrew (‘my Hebrew isn’t fully fluent’). Proficiency in English in this context seemingly meant socialising was less effortful than socialising in Hebrew, and afforded access to classmates from backgrounds more aligned with his own.

### Familial connection

Bilingualism did not only assist with integration with peers, it also facilitated greater familial connection. For example, Yoel explained praying in Hebrew alongside his father made him feel closer to him:

When I daven [pray] on holiday with my father it does, it really does [make me feel closer to him]. (Yoel, 8.5 years old, UK)

Yoel’s emphatic repetition (‘*it does, it really does*’) effectively relays his sense of deep connection to his father facilitated through Hebrew prayer. For some, understanding a second language facilitated familial integration during religious festivals. For example, Saul explained that his favourite holiday was ‘definitely’ Passover and explained why:

. . . well there’s the Afikomen [a piece of Matza traditionally hidden for children to find], but in our family we do it a bit different. Instead of the parents hiding it and the kids need to look, the kids hide it and the parents need to look. If they don’t find it then every kid gets a present. (Saul, 11.5 years old, Israel)

In this case, knowledge of Hebrew enabled understanding of and participation in a fun religious custom with a Hebrew name, alongside other young family members. The description of children hiding the *Afikomen* effectively relays the collaborative nature of this ceremony. It also seems that this variation of the ritual provided a sense of familial pride for Saul who emphasised: ‘*in our family we do it a bit different*’ (Saul, 11.5 years old, Israel).

### Religious connection and depth

Several participants referred to the religious and spiritual value of learning Hebrew in addition to English. When asked if she liked using Hebrew words, Yael responded: ‘*Yeah it gets me . . . more connected to HaShem [G-d]*’^1^ (Yael, 12 years old, UK). For Yael, it appears that Hebrew allows something more transcendent than communal religious experiences: It allows a sense of greater communion with G-d.

Many participants reflected on the spiritual connection they felt when speaking Hebrew as an additional language. Shalom explained that through speaking Hebrew, he feels a connection to his forebears. He further related: ‘*And also feels like this is the holy language and it’s the language of Israel*’ (Shalom, 9 years old, Israel). The phrasing ‘the holy language’ reflects the gravitas and profound meaning of knowing what to him is the only sacred language. The second clause of the sentence may be purposefully ambiguous to reflect that knowing Hebrew facilitates integration in modern Israel, or alternatively among the people of Israel.

Several participants reflected on the religious depth of Hebrew compared to English:

. . . it’s [i.e. Hebrew’s] more of a deep language that has connections like thousands of years old, but it’s more ancient and there’s more like every word is connected and it’s like more deep . . . (Raphael, 14 years old, Israel)

Repetition of the word ‘deep’ illustrates the profundity of Hebrew that Raphael values. It also appears that this is complemented by the ancient provenance of Hebrew. The emphasis on this seemingly reflects the considerable meaning that Raphael finds in the antiquity of the language. However, Raphael also valued knowing English, partly due to English prayerbook translations assisting him in understanding Hebrew prayers:

Last year I would read the prayers in English. Ones which I didn’t understand . . . . Now I only pray in Hebrew. I’m always saying my prayers in Hebrew but occasionally I read the translation so I can understand . . . In Hebrew that feels more like right and that’s what we’re used to . . . Feels like this also because I’m used to it and also because . . . that’s how it was meant to be said. That’s like the correct way. English is like it’s not the Sefa Kadosh [Holy Language]. It’s different from the English right?

. . . Yeah, and there’s more meaning to that. [. . .] It feels great. I’m exactly speaking the holy language, that’s something else! (Raphael, 14 years old, Israel)

Like Shalom who also referred to speaking ‘the holy language’, Raphael finds enjoyment and directly references the greater religious meaning inherent in using Hebrew to pray. The phrase ‘that’s something else’ seemingly reflects great marvel and pleasure in being able to speak this sacred language. Unlike Shalom, Raphael provided several reasons explaining why praying in Hebrew is distinct from worship in English. First, there is a sense of familiarity in praying in Hebrew (and Raphael repeats ‘used to’). A second reason that appears more important to Raphael due to his reiterating this point (‘*feels more right*’, ‘*meant to be said*’, ‘*correct way*’) is that praying in Hebrew appears more proper, the way Jewish worship was intended to be vocalised. By saying ‘it feels more right’, Raphael may be emphasising that praying in Hebrew intuitively feels more fitting than English, but in a spiritual or emotional rather than objective way.

### Scholastic importance

Several participants reflected on how Hebrew was important scholastically. As Jonah explained: ‘*That’s what I want to do . . . Yeah, I want to do Hebrew GCSEs*’ (Jonah, 12.5 years old, UK). This represents that proficiency in Hebrew as a second language has become an educational aspiration that can also be pragmatically useful as it can be counted as a national school examination. Bilingualism was also important to participate in the classroom and in the playground. Shalom explained this succinctly: ‘*At school . . . normally I speak Hebrew . . . When I’m with my friends then I speak English*’ (Shalom, 9 years old, Israel).

### Social impacts

For some, bilingualism helped foster social connection and buffered the impact of bullying:

But sometimes I do speaking in English with a friend of mine who knows English really good.

[Reason enjoys this:] . . . nobody else understands what we are saying [. . .] They trying to say something mean, I pretend . . . what they’re saying which is mean in Hebrew means something nice in English. They’re just embarrassed. (Devorah, 11.5 years old, Israel)

This highlights an unexpected social benefit of Hebrew-English ability; it can be used to deflect insults. It also appears that Devorah values the uniqueness of knowing English in a primarily Hebrew monolingual environment. A sense of mutual social connection and exceptionality is conveyed by the phraseology ‘nobody else understands’. However, Devorah valued having gained proficiency in Hebrew. She explained that she felt connected to children in her class when she sang a Hannukah song in Hebrew. She explained why:

Because . . . they’re all saying like, it was so fun, and they enjoyed it. And that I’m really good at singing and they want me to do it again . . . it just felt like getting more closer. (Devorah, 11.5 years old, Israel)

Devorah appeared to value giving others pleasure through her rendition of a Hebrew song (and also valued their acknowledgement of her talent). For Devorah, it appears she experienced a sense of heightened affinity and affection, which was engendered through the Hebrew song recital. This account reflects how gaining ability in Hebrew as a second language served as a conduit for social affirmation and engagement, which then created a sense of closeness towards her peers.

## GET: preference of bilingualism and dislike of monolingual approach

Many participants expressed they preferred to be bilingual due to its benefits and disliked the prospect of only knowing one language. Most participants reflected that Hebrew-English bilingualism provided a sense of enjoyment and empowerment.

### Religious value

Sometimes the deep value that participants placed on Hebrew-English bilingual ability was conveyed by the short yet powerful language employed. For instance, Jonah expressed how it felt to know another language (Hebrew) in addition to English: ‘*It feels like a blessing*’ (Jonah, 12.5 years old, UK). For some, the advantages of bilingualism were pragmatic and straightforward. For example, Joshua explained: ‘*I prefer to pray in Ivrit [Modern Hebrew]*’. When asked why, Joshua explained: ‘*Because: I understand the prayer in Ivrit so I’d have to try and transfer them over to English*’ (Joshua, 10 years old, UK). The language ‘I’d have to try’ seemingly is employed to emphasise the pragmatism of not trying to take this more burdensome approach.

### Pragmatic considerations

Secular and non-religious utilitarian uses of bilingual ability were also emphasised by participants. For instance, Yair explained bilingualism is ‘*. . . good, because . . . if you’ve heard . . . something big happening . . . you . . . get a more . . . in-depth article from . . . local [Hebrew] news . . . And . . . if you’re in America . . . you probably will get American news [in English]*’ (Yair, 12.5 years old, Israel). In this sense, it appears Yair values the greater agency concerning knowledge of current events facilitated by bilingualism. Kfir also valued the utility of Hebrew-English proficiency: ‘*It’s useful being able to know two languages. . .That you could understand like more stuff*’ (Kfir, 11 years old, Israel). This somewhat ambiguous statement conceivably refers to being able to access literature and words spoken in two languages, which affords Kfir two streams of information from two distinct cultures.

### Specific linguistic features

For some, knowing Hebrew was valued over only knowing English, and participants preferred to speak Hebrew due to features of the latter language. For example, Devorah, raised in an English-speaking home in Israel, explained why she valued also having learnt Hebrew in school:

Hebrew words are a lot shorter sometimes . . . So it makes it easier to read them . . . And in English . . . the silent word . . . like, it’s a lot more confusing. And Hebrew is a lot less confusing (Devorah, 11.5 years old, Israel)

The antithetical and emphatic language (‘a lot more’, ‘a lot less’) may be employed by Devorah to reflect the extent to which she experiences Hebrew as more straightforward and less mystifying than English. Relatedly, Jonah explained he prefers to use the word ‘*Shabbat*’ rather than Saturday. When asked why, he explained: ‘*Because I prefer the word, and it’s much easier to say, than saying Saturday. [Explained preference is because of:] . . . How you say it*’ (Jonah, 12.5 years old, UK). Like Devorah, Jonah found certain Hebrew words easier to enunciate. However, unlike Devorah, Jonah provided other reasons for his preference. For Jonah, there is something about the way the Hebrew word is enunciated, which means he prefers it to saying it in English. Conversely, some participants were more ambivalent, disliking some phonetic features of Hebrew but liking others: For instance, Yair explained he did not like the sharp ‘ch’ sound of a Hebrew consonant: ‘*actually, I don’t like the “chet”*’, noting that this sound also appeared ‘in Arabic and a bunch of other languages’. However there were other features of Hebrew that Yair appreciated: ‘*they [i.e. Hebrew] don’t have the* “*T-H*” *[i.e. ‘th’] sound*’ (Yair, 12.5 years, Israel).

### Empowerment and pride

A sense of pride could be engendered from knowing a language that peers did not know. Jonah (the only child in his class to speak Hebrew) provided a simple explanation of what it felt like to be able to speak to his grandmother in Modern Hebrew: ‘*Proud of it*’. Devorah explained the reasons she enjoyed speaking Hebrew:

Because knowing two languages is fun . . . Because when knowing more than one language, it makes you feel smarter. (Devorah, 11.5 years old, Israel)

Devorah’s account demonstrates the sense of enjoyment and empowerment that can be derived from Hebrew-English proficiency. It seems especially important that for Devorah, bilingual proficiency can provide a sense of intelligence, potentially due to the fact that neurotypical peers do not have similar proficiency.

A sense of pride was evident in other participants’ accounts, albeit in divergent ways. For instance, Tiffany explained she prayed in Hebrew and proudly remarked that despite not yet being fluent, she was gaining fluency: ‘*Ivrit . . . And I’m getting more fluent at it every day*’ (Tiffany, 10 years old, UK). Conversely, Kfir, who was fully fluent in English, explained he thought he was the best in his Israeli [primarily neurotypical] class at speaking English:

. . . I think the best (at speaking English) . . . [Re exams:] Yes, I’m always at least past the 95. And usually, if not, it is because I forgot something like a period [i.e. a missed full-stop] (Kfir, 11 years old, Israel)

By relating that he generally scores over 95% and typically is only marked down due to missing a full stop, Kfir may be striving to indicate that he only loses marks for relatively inconsequential ‘mistakes’ which are not reflective of his English ability. The sense of pride conveyed by this is heightened by Kfir revealing he knows his unchallenged position within the class vis-à-vis English proficiency.

### Aesthetic appreciation

Devorah explained that she has a predilection for a certain Hebrew table-hymn:

I like the one before we do Kiddush . . . ‘Shalom aleichem malachei hashalom’ . . .. That one. [In explaining why it was her ‘favourite’:] . . . Because it sounds so pretty and everyone sings it, so afterwards it sounds like a harmony. (Devorah, 11.5 years old, Israel)

Devorah’s account conveys the pleasure gained from listening to a Hebrew song within *Kiddush*, a Hebrew ritual. The repetition of ‘sounds’ in a positive way (‘*so pretty*’, ‘*like a harmony*’) conveys the pleasure gained from listening to Hebrew hymns.

### Being afforded greater choice

Saul explained what it felt like to be able to sing in two languages: ‘*It’s pretty nice. In England it’s more like one choice but in Israel it’s like one choice, I get two*’ (Saul, 11.5 years old, Israel). A sense of agency is conveyed by Saul using the word ‘choice’; furthermore, the word ‘get’ could be used to convey that bilingualism is experienced as a ‘gift’ being given to him.

### Repetition and routine

Aviva enjoyed Hebrew activity (in this case, prayer) for different reasons:

It feels nice because I know what’s going on because it’s basically the same thing I’ve been doing since I was little. So being that repetitive . . . you do research in autism you know everything’s about repetitiveness. So like . . . doing the routine of standing up, sitting down, orders . . . So it’s the same thing, the same order and the same routine as when I was . . . younger. (Aviva, 17 years old, UK)

Aviva uses language that reflects rule, regimentation, and familiarity (‘rule’, ‘routine’, ‘orders’), seemingly to emphasise the non-changing and prescribed forms of Hebrew prayer. The comprehensive importance of this for an autistic person is effectively relayed by Aviva when she explained that ‘in autism’, ‘*everything’s about repetitiveness*’. Aviva appears to use the word ‘same’ four times to convey that the Hebrew prayers feel comforting because they are intimately familiar through their ‘sameness’ and her experience of their changelessness.

### Preference for additional language beyond Hebrew and English

Interestingly, for some children with Hebrew-English ability, enjoyment was primarily derived from a third language they knew, rather than Hebrew and English. While Jonah preferred the way Hebrew words are said compared to English words, conversely, Conrad explained: ‘*I really enjoy the way German words sound*’. When asked what he enjoys about this, Conrad explained:

I like the umlauts . . . I just like the . . . lack of . . . the . . . softer sounds. ‘Cos . . . it’s . . . a harsher language . . . And, I like . . . that part of it. (Conrad, 11.5 years old, UK)

The words ‘harsher’ and ‘softer’ reflect that Conrad finds enjoyment in the distinct nature of the language. This may reflect that the unfamiliar and novel sound of German was enjoyable to him over the sounds of English and Hebrew.

### Dislike and distress over the monolingual approach

Participants expressed their distress at the monolingual approach adopted by practitioners, which they were aware existed. In general, participants indicated that schools encouraged bilingualism. However, unlike other participants, only one participant described how she was directly affected by forced monolingualism:

They didn’t allow me to do Hebrew because Hebrew is a GCSE subject . . . I did it for . . . two years of high school and then I stopped because of the GCSE . . . which is really sad because I really enjoyed Hebrew . . . (Aviva, 17 years old, UK)

Aviva effectively relays the distress she experienced due to a monolingual mindset adopted by the school, by using language that portrays the school as being restrictive (‘*didn’t allow me*’). She also relays this by explaining the difficult emotions she experienced through being prevented from studying a language she thoroughly enjoyed, merely because others deemed a GCSE as being ‘too difficult’ for her. She went on to explain her views on monolingual mindsets:

People expect me not to know so much which is a sad part because . . . I’m the same as everyone else, I just have a diagnosis from a doctor who says I’m not. That’s basically what it is. It’s just a piece of paper and like every piece of paper it can be thrown away. So it’s been very challenging to . . . well be taken seriously first of all and . . . at the beginning of the year I said I still wanted to learn Hebrew so they gave me a one-to-one but the next year they didn’t want to teach me anymore and it was upsetting to me because I wanted to learn it but you just didn’t let me. (Aviva, 17 years old, UK)

The repetition of the word ‘sad’ may be used to illustrate the injustice of assumptions made about an autistic child, which caused Aviva such a painful impact. The vivid description of the nature of an autism diagnosis seemingly reflects Aviva’s view that the ‘document’ should not encumber one’s progression. A sense of the way she regards the diagnosis as non-representative of herself is gained from her account of how the diagnosis letter can be readily discarded. Aviva reiterates the school not ‘allowing’ or ‘letting’ her continue learning Hebrew and also the affective consequence (‘really sad’, ‘upsetting to me’) to highlight the emotional impact and toll that a monolingual approach can have. A sense of this being especially egregious is gained from Aviva’s account of a presumptuous expectation of knowing less than others being the root of this.

Relatedly, Raphael asserted that any difficulty in gaining proficiency in a second language was due to his familial background and not because he is autistic.

I think that it wasn’t hard to me . . . it was just hard because of how I was raised. I don’t think learning another language is negatively affected because of autism. (Raphael, 14 years old, Israel)

By underscoring that initial difficulty with Hebrew was about the way he ‘was raised’ rather than ‘autism’, Raphael effectively relays how bilingual difficulty should not instinctively be assumed to be due to autism: Other contextual factors are important too. In fact, Raphael relayed he has now gained bilingual proficiency.

### Bilingual confusion

However, there were divergent and isolated examples of participants explaining their difficulty either with bilingualism or with gaining bilingual proficiency: Tiffany explained her difficulty with more than one language being spoken: ‘*Sometimes I get very confused. It gets very confusing with so many different languages in the house*’ (Tiffany, 10 years old, UK). The repetition of ‘very confusing’ is seemingly used to convey the difficulty of this for Tiffany. This confusion was not mirrored in other participant accounts.

## Discussion

To our knowledge, this is one of only two studies that specifically explored experiences of bilingualism from the first-person perspective of autistic children. This research study is the first to explore experiences and perspectives of Hebrew-English bilingual autistic children. Thirteen autistic children were interviewed, and strikingly rich accounts were elicited. It appeared that children were especially enthusiastic at being able to express their views on this topic, in many cases, for the first time. Two GETs were clustered from the data. The first GET reveals that overall, autistic children felt that Hebrew-English bilingualism benefitted them across multiple areas of their lives. As such, the GET ‘***Bilingualism aids religious, educational, and social integration and connection***’ is consistent with earlier findings among carers of autistic children that Hebrew-English bilingualism affords ‘multifaceted’ integration for autistic children, including religiously, socially, and spiritually (Sher, Gibson, & Browne, 2022).

The finding of a sense of pride, religious connection, and social and familial integration generated from bilingualism, alongside preferences for bilingualism among autistic children from bilingual backgrounds in England and Wales (Howard et al., 2019a; Jegatheesan, 2011), also accords with the findings of this study. This also illustrates the integration afforded for autistic children of distinct cultural backgrounds through bilingualism. As reflected by the GET ‘***Preference of bilingualism and dislike of monolingual approach***’, children reported a wide range of benefits derived from Hebrew-English bilingual proficiency, including the sense of enjoyment and empowerment derived, alongside a feeling of pride and fulfilment. This aligns well with parental accounts of the empowering nature of autistic children learning both Hebrew and English (Sher, Gibson, & Browne, 2022).

The fact that most children did gain bilingual ability and did not report any detriment in other linguistic or academic areas due to this accords well with literature which has shown that bilingualism does not cause autistic children to experience language delays and does not hinder autistic children’s language acquisition (Garrido et al., 2024; Uljarević et al., 2016). The finding that children in this study were supported by schools to gain bilingual ability also aligns with previous research showing the Jewish community diverges from other communities in promoting Hebrew-English bilingualism for autistic children (Sher, Gibson, & Browne, 2022). It also accords with literature showing positivity towards bilingualism in certain UK bilingual families and schools (Digard et al., 2023; Sher, Gibson, & Browne, 2022) and mirrors the finding that greater encouragement of bilingualism exists within multilingual settings (Howard et al., 2024). Previous research has revealed that parents and educators of autistic children have found that autistic children were frequently more adept at gaining Hebrew-English bilingual ability than their neurotypical peers (Sher, Gibson, & Browne, 2022). It is conceivable that this explains the participants’ very positive outlook on Hebrew-English bilingualism in this study, and it is possible that this positivity may not be mirrored to the same extent among neurotypical peers. Certainly, it seems clear that the finding of an autistic child (Aviva) being prevented from attending Hebrew classes because it was deemed the language would be ‘too hard’ for her to succeed at GCSE level appears related to autism specifically, and may not be mirrored in neurotypical contexts. Indeed, wider literature reveals that a lack of knowledge about autistic children gaining bilingual ability means that frequently, autistic students are told to stop attending lessons on different languages (Essex & MacAskill, 2020).

The finding that autistic children value Hebrew-English bilingualism and view it positively augments previous findings that parents and educational practitioners within the Jewish community also view Hebrew-English bilingualism positively, and are largely supportive of it (Sher, Gibson, & Browne, 2022). The fact that the value of bilingualism in enhancing relationships with family and extended family was less prevalent in this study may reflect differing priorities of parents and educators compared to children. However, it is also conceivable that autistic children placed less emphasis on this because several children were immigrants to Israel with siblings who had adjusted to learning Hebrew at distinct ages. The utility of bilingualism in promoting family connectedness may thereby have been reduced. As Yair (originally from the US) explained, his siblings spoke Hebrew at home but he spoke English at home: ‘*they speak . . . Hebrew at a high fluency because they well . . . like, grew up here much more*’. It may also be the case that, unlike other minority contexts (e.g. Kremer-Sadlik, 2005), the families of children interviewed were generally capable in both Hebrew and English. This may reflect the societal emphasis on learning English in addition to Hebrew in Israel, and the communal emphasis on knowing Hebrew in addition to English within the Jewish diaspora (Glinert, 2017; Or & Shohamy, 2017). Familial bilingual ability may have meant that bilingualism’s value did not always facilitate considerably greater opportunity to communicate with extended family in the way that it could in cultures where some extended family only spoke one community language, and not English (Kremer-Sadlik, 2005).

The fact that participants were from two distinct bilingual contexts – Israel and the UK – is a strength of this article. Interestingly, Howard et al. (2019a, 2024) found that in more multilingual settings (e.g. in Welsh settings), bilingualism was viewed more favourably by autistic children than autistic children situated in more monolingual settings (e.g. schools in England). However, this finding was not mirrored in this study, as both children from more monolingual Israeli settings and those from UK settings viewed bilingualism positively. This may be due to geopolitical contexts influencing autistic children’s perspectives on bilingualism. For example, in Israel, the dominant influence of the United States (US) on Israeli culture and Israel’s strong financial, cultural, and diplomatic ties with the US has made English a desirable language that children are encouraged to learn (Or & Shohamy, 2017). In Israeli Hebrew-medium schools, English is the first additional language that children are taught, often from age 7 onwards (English becomes mandatory from age 9; Or & Shohamy, 2017). There is also evidence that parents strongly encourage their children to gain proficiency in English, which is viewed as an asset promoting social mobility (Or & Shohamy, 2017). Relatedly, in the diaspora, Hebrew is viewed as an important language for ensuring Judaism’s continuity and is prized for its religious and cultural significance, alongside its role in fostering connection to Israel (Gross & Rutland, 2022; Mintz, 1993; Schiff, 1998). As such, almost all Jewish schools in the UK provide at least some instruction in Hebrew (Miller, 2011). This may explain why both participants in the UK and Israel valued bilingualism to similar extents.

## Implications

Given the higher levels of peer victimisation experienced by autistic children (Cappadocia et al., 2012; Sterzing et al., 2012), which are linked to adverse educational outcomes (Adams et al., 2016), the findings that bilingualism facilitated closer relationships to peers and enabled deflecting peer victimisation appear particularly significant. It seems appropriate that these positive potential outcomes of bilingualism should be more widely known, so that this protective impact can be more widely fostered. Similarly, public stigma towards autistic children is prevalent in schools (Aubé et al., 2021) and is experienced by autistic children in all cultures, including Jewish children, although efforts have been made more recently to reduce this stigma within Jewish communities (Sher, Gibson, & Sher, 2022). The findings that Hebrew-English bilingualism promoted autistic children’s agency and pride and enhanced a sense of empowerment are significant. These findings could be amplified and harnessed in classroom and home settings to complement the impressive progress made to eliminate stigma towards autism within the Jewish community (Sher, Gibson, & Sher, 2022).

Given the widespread propensity of practitioners to caution against autistic children gaining bilingual proficiency (Beauchamp & MacLeod, 2017; Kay-Raining Bird et al., 2016) despite a lack of empirical evidence for this (Uljarević et al., 2016), knowledge of autistic children’s views should be more widely disseminated. It seems essential, therefore, that practitioners who wield power and influence in relation to guiding parents’ and schools’ linguistic choices for autistic children (such as early years practitioners, educational psychologists, paediatricians, speech and language therapists, and teachers) should be made aware of the potential for detrimentally curtailing children’s social, religious, and cultural opportunities by adopting a monolingual approach. Training for practitioners that includes details of research which highlights this negative impact on autistic children is one obvious next step. Providing practitioners with such information has been previously advocated (Davis et al., 2021). It is also crucial that autistic children’s voices are heard in all such decision-making. The findings of this study indicate that autistic children prefer bilingualism to monolingualism and that some believe autism should not be the primary consideration in bilingual decision-making. While this study focussed on one bilingual context, it complements a growing body of research now showing that in the experience of autistic children and their carers, bilingualism enriches and enhances autistic children’s experiences across multiple domains, including their social, educational, communal, and/or religious lives (Howard et al., 2019a; Jegatheesan, 2011). Practitioners must take the views of autistic children into account when making decisions that directly affect them.

There were isolated accounts of children being prevented from gaining Hebrew-English bilingualism. The finding of emotional distress caused by this monolingual approach complements previous literature arguing that the adoption of monolingual approaches for autistic children is pervasive (Beauchamp & MacLeod, 2017; Kay-Raining Bird et al., 2016), and that the monolingual approach risks abrogating autistic children’s basic rights (Gréaux et al., 2020). Overall, the potential for causing distress by limiting an autistic child’s linguistic repertoire through monolingual approaches needs to be a weighty consideration when practitioners provide advice on this topic.

## Strengths, limitations, and future research

Strengths of this study include the novelty of the topic explored, and successfully eliciting detailed responses from a marginalised population whose voice is frequently unheard. For the first time, Jewish autistic children expressed their views on Hebrew-English bilingualism, which occupies a central role in their everyday lives. Moreover, the study gathered data from autistic children from a wide range of backgrounds, ranging from non-observant to strictly-Orthodox. This means the study is not limited by the ‘research parochialism’ of only exploring the experiences of a very small subset of a population (Cohen et al., 2018; Smith, 1975). This study does have several limitations. Four out of 13 participants were female. Seven out of 13 participants resided in the UK, and six lived in Israel. It is also likely that this study does not reflect autistic children who are less able to express their thoughts as clearly as the participants presented theirs here. Similarly, support for bilingualism experienced by participants may be partly attributable to the generally high level of verbal ability that participants exhibited in interviews. It is conceivable that support for bilingualism may not have been mirrored in contexts where participants had lower levels of verbal ability. Notably though, IPA does not seek generalisability or replicability. Instead, it aims to achieve ‘theoretical transferability’ (Smith et al., 2022, p. 45). This allows revealing ‘core constructs’ of phenomena, which can then inform practical psychological or clinical application (Smith, 2004, p. 51).

The fact that approximately half of the participants were from the UK and half from Israel may mean that findings do not reflect the experiences of children from one country. Future research could explore the bilingual experiences of autistic children within each of these settings in greater depth. Another avenue for further research could include exploration of autistic children’s bilingual experiences across several other cultures and communities. In this way, the voice of autistic children from diverse backgrounds may be further elevated.

## Notes

1. Names to denote G-d are abbreviated in this article (e.g. G-d, A-mighty) to accord with Jewish religious practice of not writing G-d’s Name in full. One reason for this is to avoid G-d’s name being upon paper which could be disrespectfully discarded, ripped or destroyed (see Steinmetz, 2005, p. 2).
